# Hierarchical molecular tagging to resolve long continuous sequences by massively parallel sequencing

**DOI:** 10.1038/srep01186

**Published:** 2013-03-08

**Authors:** Sverker Lundin, Joel Gruselius, Björn Nystedt, Preben Lexow, Max Käller, Joakim Lundeberg

**Affiliations:** 1 Science for Life Laboratory, KTH, Gene Technology, Solna, 171 65, Sweden; 2 Science for Life Laboratory, Stockholm University, Department of Biochemistry and Biophysics, Stockholm, 106 91, Sweden; 3 LingVitae AS, Husøysund, 3132, Norway

## Abstract

Here we demonstrate the use of short-read massive sequencing systems to in effect achieve longer read lengths through hierarchical molecular tagging. We show how indexed and PCR-amplified targeted libraries are degraded, sub-sampled and arrested at timed intervals to achieve pools of differing average length, each of which is indexed with a new tag. By this process, indices of sample origin, molecular origin, and degree of degradation is incorporated in order to achieve a nested hierarchical structure, later to be utilized in the data processing to order the reads over a longer distance than the sequencing system originally allows. With this protocol we show how continuous regions beyond 3000 bp can be decoded by an Illumina sequencing system, and we illustrate the potential applications by calling variants of the lambda genome, analysing TP53 in cancer cell lines, and targeting a variable canine mitochondrial region.

High-throughput sequencing instruments have continued to demonstrate increasing number of DNA bases decoded per run^1^,^2^,^3^,^4^,^5^. However, despite the tremendous success the new generation of sequencers have had in terms of cost and sequence throughput, the length and accuracy of the traditional Sanger based sequencers have remained a commonly accepted standard for validation^6^,^7^. In parallel, there has been a gradual shift in interest from single base pair variations, to copy number variation, structural variation and de-novo assembly approaches, increasing the need for long, high-quality sequences^8^,^9^,^10^,^11^,^12^. Shorter reads have difficulty mapping to low complexity regions and resolving variation involving the orientation of larger regions. Furthermore, the ability to phase variations directly from the data is relevant for many biological questions where haplotype information is unknown or limited. Phasing directly is limited by the read length of the sequencing system^13^. Short reads also pose great challenges for de novo assembly, although many algorithms for this problem have been developed^14^, they struggle to completely assemble even small bacterial genomes^15^.

To overcome the problems of short reads, protocols for linking two reads together have been developed^10^,^16^. Cloning-free methods for linking reads together over long stretches of DNA range from 2 to 20 kb with increasing need for input DNA for increasing insert sizes. Cloning-based libraries such as fosmid libraries can further increase the insert size^17^. By incorporating a known distance between two sequence reads during construction of the library, that information can be utilized when deciphering the structure of a known genome, ordering the contigs built from assembly, to span or aid mapping in low complexity regions. The drawbacks of these protocols are the consumption of vast amounts of DNA as well as laborious and manual steps limiting scalability and robustness. Further, although structural problems equal to or below the insert size of long insert libraries can theoretically be resolved, the gain for phasing remains limited since the sequence between the linked reads is unknown. For this reason, phasing often relies on a high polymorphism rate of the particular genome in order to link the variation^15^. Recently, the use of randomized nucleotides have been reported in conjunction with massively parallel sequencing, in order to improve the quality of detection^18^,^19^,^20^,^21^. These studies have been based on short target amplification, and show the usefulness of randomized tagging to count the number of unique molecules. The usefulness of nested short-read libraries and tag-directed local assembly have been shown previously but has been limited in target length to 500 bp^22^. Very recently a long fragment dilution protocol have been used to enable whole genome haplotyping applicable for SNPs in human genomes^23^. Despite these advances, decoding a long continuous sequence remains a challenge.

The procedure described in this report, Tile-seq, is a targeted PCR-based approach that can aid in structuring massively parallel sequencing reads and help low complexity spanning, but also links information over the whole target region thereby increasing the information obtained. This is achieved by spreading the shorter reads of the sequencing system over the entire region, while keeping them indexed for their molecular origin. Also, the information of eight insert sizes is incorporated rather than one. Since the larger regions are indexed, the mapping or assembly is a local problem, and the varying insert size information provides help to resolve low complexity regions and structural variation. The outlined protocol for multiplex long range analysis supersedes that of traditional Sanger sequencing in terms of the regional length and complexity possible to decode, and also has potential for great accuracy or dynamic range in detection. Further, we show the principal utility of using randomized nucleotide tagging to index a large pool of molecules for quantifying and linking unique reads together.

## Results

**Figure 1:**
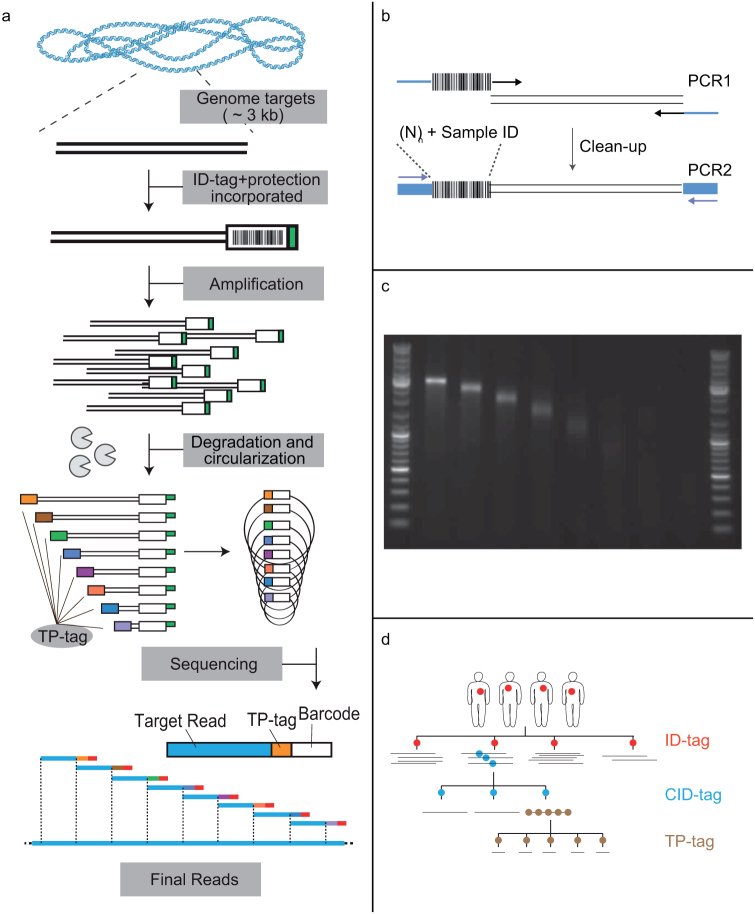
Tile-seq basic procedure. (a) Genomic DNA is targeted and amplified to include molecular barcodes. The barcodes are protected and subsequently exonuclease degraded. The reaction is sub-sampled and stopped at distinct intervals, and indexed with a tag specific for the time of sampling. The molecules are circularized, fragmented and enriched for the junction between degraded and indexed ends. The junctions of degraded and indexed ends are amplified and sequenced. (b) Representation of the two PCRs incorporating a randomized clonal ID sequence and a known sample ID sequence. (c) Example agarose gel of eight samplings during exonuclease degradation of an amplicon showing the decreasing pool length of each TP. (d) The hierarchical structure given by three incorporated tags, sample id-tag (ID), clonal ID-tag (CID), and time point-tag (TP).

To show the general principle of the protocol, the lambda genome was targeted with 19 PCR amplicons. Each amplicon was indexed at one end of the amplicon (ID-tag) and amplified in a two-step PCR process (first ID incorporation, then amplification). The end containing the index was protected by introducing a 3′ ssDNA overhang. The amplicons were exonuclease degraded leaving the ID-tag intact, sub-sampled at eight different time points (TP1-8), circularized, and sequenced (Figure 1a). To show potential applications we targeted the TP53 gene (exon 2 – 9) in one continuous 3184 bp region in four cell lines. Four different ID-tags were included to distinguish four different sample origins (NA10831, U251, U2OS, A431). We also targeted one highly variable, low complexity region in the canine mitochondrial genome. For the applications an additional randomized index was included and used for clonal identification (CID-tag) (Figure 1b and d).

### Lambda genome

**Figure 2:**
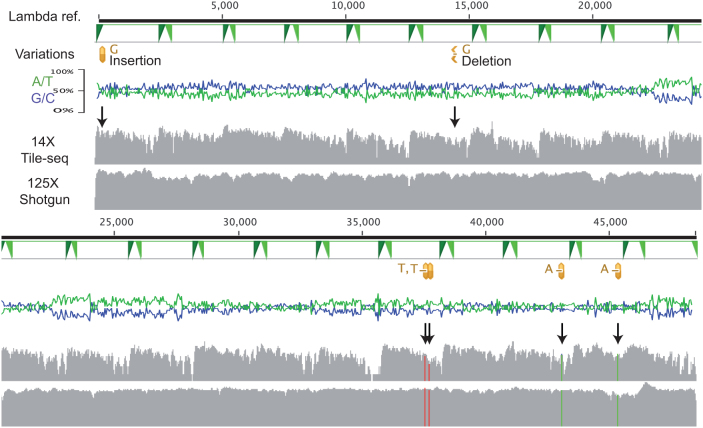
Lambda genome amplicons. Top: a schematic representation of the lambda genome and primer sites for the 19 amplicons in green triangles. The protected end was incorporated at the left side of these amplicons. The variations detected by sequencing the lambda genome (both by shotgun and Tile-seq) are shown in yellow below the reference. Middle: base composition in 20 bp sliding window (G/C blue, A/T green). Bottom: Tile-seq and reference shotgun coverage with the variants highlighted with arrows. The reference and results are displayed over two rows.

From the lambda genome six differences to the Genbank entry were detected by shotgun sequencing (Figure 2). We covered the genome using Tile-seq at 99.8% (93% with 5x) across the different amplicons. Due to circularization bias, the protocol produces lower coverage in the 3′ end of each amplicon (i.e. the longer fragments). The sequence depth recovered in this experiment was on average 14× for the entire genome providing acceptable variation of coverage (Figure 2). If high coverage in the 3′ end were necessary, a separate PCR with reversed primer design would incorporate the protecting uracil in both ends. Some of the potential strengths of this method compared to traditional capillary sequencing, apart from longer analysis regions, are accuracy and resolution of variants. At a 5× depth cut-off, all of the differences detected by shotgun were identified without false positives (Figure 2). This shows the ability to pool long-range amplicons and call simple mutations at 14× average coverage, spanning a 50 000 bp region.

### Human targets

**Figure 3:**
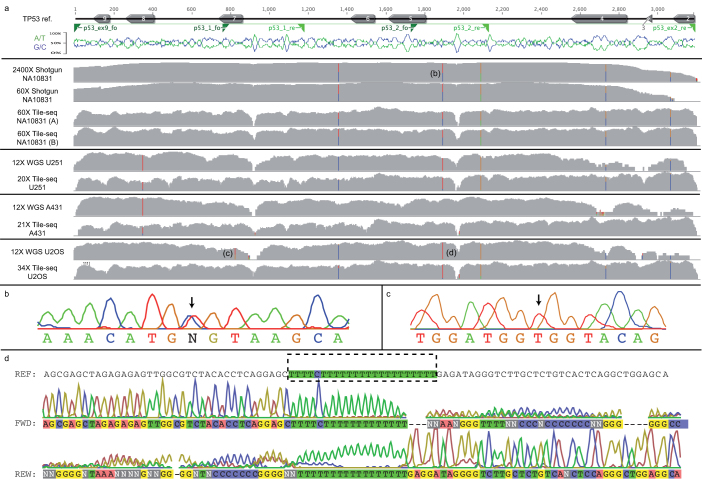
TP53 amplicon results. (a) Top: shows schematic representation of the amplicon spanning exon 2 to exon 9 (right to left) and primer sites for Sanger sequencing. The protected end was incorporated on the right side for this amplicon (exon 2). Base composition is shown in a 20 bp sliding window (G/C blue, A/T green). Coverage plots are shown in four parts, one for each sample, with the corresponding reference sequencing (Whole Genome Shotgun (WGS) for cell lines and PCR shotgun for NA10831). Variants are highlighted by colour, and positions referring to panel b–d are indicated in parenthesis. (b–d) Three Sanger validated regions, showing agreeing calls for Tile-seq (b and c, arrows) and a general difficulty in determining homopolymers (d, dotted box).

**Figure 4:**
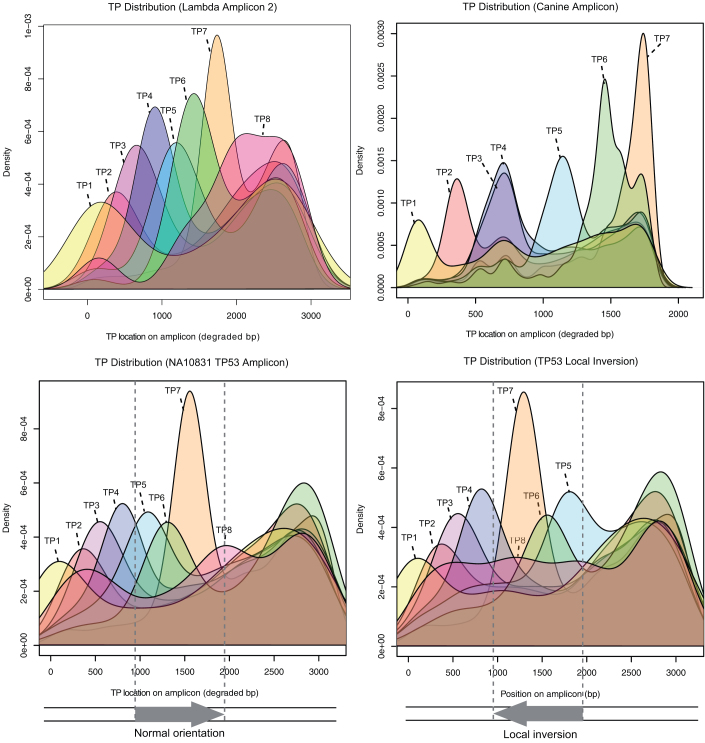
Time-point tag occurrence after mapping to the references. The mapped position of each respective TP-tag detected of each amplicon is plotted using a Gaussian kernel density approximation (R). Top left: lambda amplicon 2. Top right: the mitochondrial canine amplicon (TP8 omitted for canine amplicon due to the shorter amplicon length). Bottom left: the NA10831 amplicon. Bottom right: the NA10831 data mapped to a locally inverted TP53 reference sequence, illustrating the possibility to detect structural variation from TP-tag occurrence.

A long range PCR towards TP53, exons 2–9, was setup and used to amplify four different targets; a reference sample: NA10831, and three cancer cell lines: U2OS, U251, and A431. The reference sample PCR product was also sequenced using an amplicon shotgun approach, and the cell lines had been shotgun sequenced to average genome coverage of approximately 21× for U2OS and 15× for U251 and A431. For the TP53 region targeted in this study the average coverage for the shotgun cell line data was approximately 12x. Tile-seq yielded 60×, 34×, 20× and 21× respectively for the four targets. Generally the shotgun data and the Tile-seq data concurred. For NA10831, Tile-seq detected heterozygous variants with excellent correlation to the stochastically sheared amplicon (Figure 3). In this particular case, the stochastic shearing produced lower coverage at exon 2 and 3 for the amplicon, where Tile-seq had reasonably even coverage across the region (Figure 3a). One heterozygous position was also verified by Sanger sequencing (Figure 3b). For U2OS a triple thymine heterozygous deletion was supported by two high-quality reads in the shotgun data, not found in the Tile-seq data. Another variation based on seven good quality mappings was a heterozygous T/G (4 and 3 reads respectively, Figure 3a, 3c), which was not found in the Tile-seq data based on 26× coverage. Sanger sequencing supported the Tile-seq call (Figure 3a, 3c) and likely the shotgun variants were errors from the low coverage of the region (three reads supporting the variant). The Sanger sequencing of the homopolymeric region could not resolve the dispute (Figure 3a, 3d) but in general show the disrupting effect of homopolymers for traditional sequencing. This illustrates the potential for using the described protocol for validating WGS variations across long distances containing difficult regions. Each time point tag (TP-tag) should approximately correlate to a certain region of mapping to the amplicon. A clear trend following the degradation can be seen, albeit with some background trailing towards shorter molecules (Figure 4, Supplementary Figure S2). We believe the common peak around the end of the amplicon could be residual activity of exonuclease III in the S1 nuclease buffer. Although this activity should be limited, the circularization of shorter molecules should be favoured, causing the common short molecule peak. When the reference was modified to include a simulated 1 kb inversion, the information supplied by the TP-tags clearly indicated a structural discordance to the data obtained and the reference used (Figure 4).

### Mitochondrial sample

**Figure 5:**
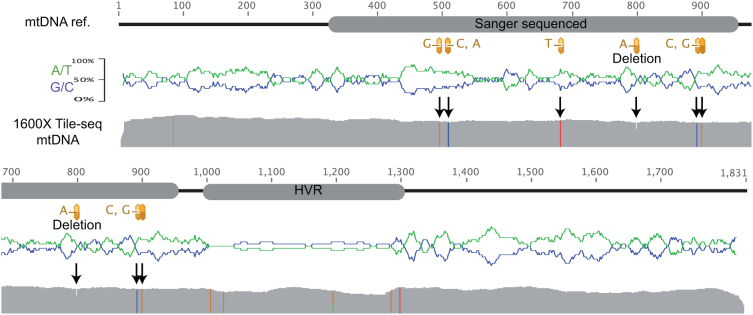
Mitochondrial canine amplicon. Top shows a schematic representation of the amplicon. Left grey box indicate the region validated by Sanger sequencing and the variants detected shown below in yellow, right grey box show the highly variable repeat region (HVR). The protected end was incorporated on the left side of this amplicon. Middle: base composition in 20 bp sliding window (G/C blue, A/T green). Bottom: Tile-seq coverage with agreeing variants to Sanger calls (arrows), and additional non-validated variants by coloured columns in the coverage plot. The reference and results are displayed over two rows.

Sanger sequencing revealed seven variants of the amplicons, all detected by Tile-seq (Figure 5). The highly variable repeat region could not be resolved completely by assembly or mapping, although certain positions seems to share common variants (Figure 5). As with homopolymers the low complexity highly variable region does not disrupt the calling of variants before or after it. Coverage for the mtDNA sample was the highest (1600×) but CID-redundancy was not enough to allow phasing. With an increased redundancy of barcodes in the final libraries clonal assembly for highly variable regions and phasing of haplotypes could be feasible.

## Discussion

Chimeric constructs, most likely formed during the library PCR where each construct share a common hairpin circularization adapter (see discussion in supplementary material), are common in the raw data (~50%) but are reduced from filtering on agreeing TPs (joined during circularization). We have identified a slight risk of false positives when only running one amplicon from multiple samples in parallel, this due to the chimeras introduced during library PCR. In the experiments described above the amplicon diversity in pooling did not present this type of limitation. Potentially alternate polymerases for library amplification that could improve on this issue, as well as alternate adapter strategies for circularization. Although efficient, the transposase-based library amplification also introduced some bias towards the library adapters (Supplementary Figure S3 and S4) which is worth mentioning for similar protocols seeking to take advantage of this highly efficient way of constructing libraries (also see discussion in supplementary material). With this gained knowledge, the authors wish to suggest that adapter sequences be designed with as large edit distance to the transposase recognition as possible.

In this study, we report on a procedure for analyzing targeted regions of a genome. In principle, the procedure could also be applied on shotgun libraries where the tagging and amplification strategy would need to be slightly modified. The CID tag will for this approach be used as the ID-tag, i.e. for identifying the target region. The challenge of a shotgun library is the extreme level of multiplexity (1.5e11 molecules for 0.5 μg of 3 kb DNA) and the subsequent need for protocol efficiency and sequence depth.

The least efficient step of the protocol is the circularization step. We show the adaptation of the protocol for 3 kb inserts, which is a relatively good yielding insert length commonly used by standard mate pair protocols. Since exonuclease III is not directly limited by DNA length, one could envision increasing the inserts to tens of kilo bases. The loss of yield in the circularization step can be compensated by increasing starting material, which however would require a higher number of CID-tags, and consequently sequencing depth to cover them. Even for the setup at hand the number of CID-tags incorporated require substantial sequencing depth to be covered multiple times, but longer inserts may be possible if coupled to an increased amplification efficiency to create more of each tag prior to circularization. This could potentially be realized by alternative amplification strategies such as multiple displacement amplification or cloning, or by favouring intra-molecular ligation by solid support or emulsions to increase efficiency.

Previous library protocols have been able to reconstruct 500 bp continuous fragments from Illumina sequencing based on concatemerization and random shearing^22^. The dependence on concatemerization restrains the target length possible to reconstruct in this way. The continued development of Illumina sequencing chemistry has now reached 2×250 bp using a MiSeq instrument and therefore made this range of continuous sequence reads more easily attained. The existing technologies for long reads remain traditional Sanger sequencing, and more recently introduced Pacific Biosciences (PacBio) SMRT sequencing. Tile-seq handles 3 kb fragments, thereby producing the longest most accurate continuous reads currently available. PacBio SMRT sequencing on average produce 2 kb reads with 85% accuracy, and the technology is not as commonly available as Illumina.

Tile-seq could be applied to finishing of de novo genome assemblies by simultaneously target a large number of assembly gaps formed e.g. during scaffolding with a 3000 bp mate pair library, ultimately filling each gap by local assembly of the Tile-seq data. In the same way structural variation breakpoints could be resolved from primer design influenced from the original detections. Moreover, being able to extend the target region from a few hundred base pairs as in 454 amplicon sequencing to 3000 bp in Tile-seq could provide a major improvement of the throughput and efficacy of phylogenetic and taxonomic studies. Presently the protocol is limited in sample throughput, but given the extreme sequence throughput of the sequencing systems available the protocol is still competitive. Sample preparation for the protocol described here is automated and high throughput, with the throughput-limiting step being the PCR. After PCR the samples are pooled and the procedure scale with the throughput of the sequencing instrument. Likewise, the major impact on cost for the library preparation is determined by the PCRs and not by subsequent ligation steps. This because of the protected ID-tag that enable direct pooling after PCR. In theory this protocol should be able to multiplex 50,000 amplicons per Illumina HiSeq 2000 lane at high accuracy (100× coverage, Supplementary Table S1). With the data we present in this report, we estimate a feasible degree of multiplexing to be 100–200 representing about 0.1% of the theoretical throughput (Supplementary Table S1). A Tile-seq setup to sequence 96 amplicons with a length of 3000 bp would require almost 9 times less resources for an average of 40× coverage per amplicon, as compared to Sanger sequencing stretches of 600 bp in each direction (Supplementary table S2).

In conclusion, we report a protocol for controllable degradation and tagging of pooled genomic long-range targets, in order to produce barcoded libraries suitable for short-read sequencing. Exonuclease III has previously been used for cloning experiments in combination with Sanger sequencing, but not been setup for a cloning-free system adapted for massively parallel sequencing. We have shown that with a regular PCR approach tags can be incorporated to in multiple layers of information to supply target origin, molecular origin and positional origin. By utilizing these indices, virtual read lengths equal to the lengths of the targets can be achieved, surpassing that of traditional Sanger sequencing as well as the capacity of spanning difficult region. We have showed PCR based tagging of nineteen 3000-bp targets representing the lambda genome. Viewed as an improvement of read length, this constitutes a 30-fold improvement to the Illumina system used, and illustrates the principle of converting short-read technologies into long-range analysis. We also illustrated potential applications by including a variable target region of canine mtDNA as well as the major part of the TP53 gene for cancer cell lines that, in principle, could greatly improve either accuracy or dynamic range of detection. Further, we show that the protocol is particularly suited for low complexity regions and could potentially be used as a validation platform for indels and structural variants.

## Methods

### DNA sample preparation

As input DNA three different origins were used, lambda DNA (NEB), canine mitochondrial DNA^24^ and human cell line DNA. The lambda genome was used as a control reference for no variation. The cell line DNA, U-2 OS (ATCC-LGC), U-251MG (Prof. Bengt Westermark, Uppsala University) and A-431 (DSMZ), had been obtained previously and shotgun sequenced (Akan, P., et al. manuscript submitted). DNA from the NIGMS Human Genetic Cell Repository, ID NA10831 (Coriell Institute), was used as human reference material.

### PCR and index protection

Each target was amplified by a two-step PCR, and received a distinct index included in one of the inner primers (Figure 1b, Supplementary Table S3 and S4). The indices have been described previously^25^,^26^, as well as the primers for TP53 exon 2 and 9^27^ and the general primers for TP53 and Lambda^20^. Inner cycling, 94°C 5 min, [94°C 30s, 60°C 120 s, 72°C 3 min] × 10 cycles, 72°C 5 min, 4°C hold, outer cycling the same but 30 cycles. Starting material for each PCR was approximately 200,000 molecules (assuming 500 mitochondria per cell for canine sample). After the first PCR the samples were automatically purified using polyethylene glycol precipitation and magnetic bead capture as we described previously^28^. Outer cycling was performed with general handles incorporated from PCR1, but including a uridine modification in the indexed end. The modification was used after PCR2 by incubating 1 U USER (NEB) per μg input DNA for 30 minutes at 37°C, thereby protecting the indexed end from exonuclease degradation.

### Exonuclease III degradation

For robust performance an MBS Magnatrix 1200 (NorDiag) automatic workstation was used to perform the enzymatic steps involved in degradation and sub-sampling of the libraries. The exonuclease III degradation procedure was motivated by previous published work^29^,^30^. Exonuclease III, 400U, (Promega) was incubated at 1× of the supplied buffer of the manufacturer in 40 μl at 22°C, and 5 μl were sampled for each time point. Time point 1 was mixed with the enzyme and directly sampled (incubated ca 20s during mixing), and the subsequent intervals between the time points were 185 seconds. The sampling interval was chosen by degrading a PCR product of a three kb lambda amplicon and determining the approximate length by agarose gel electrophoresis for different timed intervals (2.1 bp/s was the approximate speed determined, Supplementary Figure S1). The exonuclease reaction was stopped adding 15 μl S1 nuclease reaction buffer, including S1 nuclease 100 U/reaction (Promega). The salt concentration will inhibit further exonuclease III activity, and the reaction was kept cooled until sampling was complete. The samples were heated to 37°C for 30 minutes, where S1 nuclease removed the remaining protruding ends from exonuclease III degradation. The reactions were stopped by adding 5 μl stop buffer to a final concentration of 5 mM EDTA. S1 nuclease and exonuclease III were then heat-inactivated at 70°C for 20 minutes.

### Circularization

After degradation the samples, now eight time points (TPs), were kept separate and prepared for circularization using an adapted protocol from the literature^10^. This protocol was also automated using the MBS Magnatrix 1200 automatic workstation to facilitate rapid processing. Each DNA manipulation step was followed by a PEG precipitation with bead capture as we have reported previously for preparing libraries for massive sequencing^28^,^31^. End polishing in 1× T4 DNA ligase buffer, with 0.2 mM dNTPs, supplemented with an additional 0.1 mM ATP, 5 U T4 DNA polymerase, 10 U T4 polynucleotide kinase, and 5 U Taq DNA polymerase (Fermentas). Methylation in 1× EcoRI methyltransferase buffer, with 320 μM SAM and 80 U EcoRI methyltransferase (NEB). Adapter ligation in 1× quick ligase buffer, with 5 pmol hairpin adapter corresponding to that time point (Supplementary Table S5) and 2 μl quick ligase (NEB). Exonuclease treatment in 1× of NEBuffer 4 with 10 U lambda exonuclease, 40 U of exonuclease I and 20 U of T7 exonuclease. EcoRI digestion in 1× NEBuffer 2 with 20 U EcoRI-HF (NEB). All the above steps were performed in 50 μl volume and purified by PEG/CA purification to 20 μl. TPs were pooled and circularized in 1× NEBuffer 4, with 0.1 mM ATP and 5 μl quick ligase for 16h and QIAquick (QIAgen) purified to 60 μl. Exonuclease treatment 40 U exonuclease I and 40 U of lambda exonuclease for 30 minutes at 37°C, and 20 minutes at 80°C, then purified by MinElute (QIAgen) to 15 μl EB.

### In vitro transposition, bead enrichment and amplification

The circularized constructs were fragmented by in vitro transposition (“Nextera”, Epicentre) as described earlier for standard shotgun libraries^32^. All material from the previous step was incubated in the HMW buffer (Epicentre) in 20 μl at 55°C for 5 minutes according to the manufacturer's instructions. The samples were then purified using QIAquick columns (QIAGEN) to 50 μl in EB (QIAGEN). 25 μl M270 Streptavidin Dynabeads (Invitrogen) were washed and resuspended in 50 μl bind and wash buffer (“BW”, 5 mM Tris/HCl, 0.5 mM EDTA, 1 M NaCl pH 7.5). The 50 μl fragmented material was added and incubated for 30 min with agitation at room temperature. After binding the beads were washed 2 × 200 μl in BW, and 2 × 500 μl in EB. The beads were resuspended in 20 μl EB. PCR amplification was performed using all beads and Nextera compatible primers (Supplementary Table S6) at 200 nM and 1× Phusion HF Master Mix (Fermentas) at 72°C for 3 minutes, 95°C for 30s [95°C for 10s, 62°C for 30s, 72°C for 3 min] × 15 cycles, 4°C hold.

### Sequencing and data processing

The libraries were sequenced on an Illumina HiSeq 2000 instrument using standard clustering and sequencing reagents according to the manufacturers instructions for paired end 101 bp sequencing. The sequences were processed using custom made python scripts. First all sequences containing an ID-tag and a CID-tag were classified by writing an identifier to the sequence name. Sequences not containing an ID-tag were removed. Second, all sequences containing two or one correct TP-tag were classified and moved on, sequences with non-matching TP-tags (chimeras) or unknown TP-tags or sequences missing the circularization adapter were discarded. Third, all reads where split into a file according to the ID-tag classification. All files were mapped to the respective amplicon reference (GRCh37 for human, Genbank accession U96639.2 for canine mitochondrion, J02459.1 for Lambda). A script for duplication removal was written for this type of data where not only common mapping positions is prevalent but also common CID-tags. In the case where reads for a particular mapping position had identical read length and CID a duplication was classified, in which case the read with highest mapping score was kept, the others discarded. A local inversion structural variation was included by inverting position 939–1950 of the TP53 amplicon reference.

### Reference sequencing

The lambda genome had previously been prepared for a mate pair library according to the Illumina standard protocol for mate pair sequencing. The shotgun reads of this library were extracted for comparison in this study. Sample NA10831 was PCR amplified and fragmented using Covaris to approximately 400 bp, then prepared for sequencing using the Ovation SP Ultralow System (NuGen) and sequenced on a MiSeq (Illumina) 2 × 151 bp, using standard reagents according to manufacturer's specifications. Sample NA10831 was also Sanger sequenced using an ABI 3730xl (Life Technologies) on PCR products from primers targeting homopolymers (Figure 3, Supplementary Table S7). The canine mitochondrial sample was previously prepared and Sanger sequenced^24^,^33^.

### Data visualization

Data were viewed and analyzed in either Geneious v.5.6^34^ or IGV 2^35^. Coverage plots and variations were called and settings are described as they are presented.

## Author Contributions

S.L. performed laboratory work, data analysis, wrote the main manuscript and prepared the figures. P.L. conceived the method. S.L. and J.G. developed and optimized the methods. J.L. and M.K. supervised and directed the laboratory work. J.L. and B.N. supervised and directed the data analysis. All authors reviewed the manuscript.

## Supplementary Material

Supplementary Information Supplementary Material
