# Supplementary material for: Hierarchical molecular tagging to resolve long continuous sequences by massively parallel sequencing
Source: Sci Rep. 2013 Mar 8;3:1186. doi: 10.1038/srep01186 (PMC3592332; doi:10.1038/srep01186)
Supplement: Supplementary Information — Supplementary Material [file srep01186-s1.pdf]

Supplementary material

Hierarchical molecular tagging to resolve long continuous sequences by massively parallel sequencing

Sverker Lundin[1], Joel Gruselius[1], Björn Nystedt[2], Preben Lexow[3], Max Käller[1], Joakim Lundeberg[1,*]

[1] Science for Life Laboratory, KTH, Gene Technology, Solna, 171 65, Sweden

[2] Science for Life Laboratory, Stockholm University, Department of Biochemistry and Biophysics, Stockholm, 106 91, Sweden

[3] LingVitae AS, Husøysund, 3132, Norway

* to whom correspondence should be addressed.

Supplementary discussion

Chimeras

We detected unexpectedly high levels of chimeras when we examined the TP-tag junctions in the raw data. Our first hypothesis was that they were caused by intermolecular ligation during circularization. We lowered amounts and compared to blunt end ligation and cre recombinase based systems using data from another study (Akan P., et al, manuscript submitted). In this study blunt-end ligation circularization had a chimera frequency of around 2% and cre recombinase < 1%. Further the fragment concentration during circularization did not affect the chimeric frequency found in this protocol. We therefore concluded that the circularization was not the major contributor of this phenomenon. We suspect that the library PCR causes the elevated levels of chimeras, with the hypothesis being that the secondary structure of the circularization hairpin obscures the activity of the polymerase, causing it to stall or dislodge.

Bias of the transposase

Some interesting aspects of the transposase based library preparation (Nextera, Epicentre now Illumina) was discovered in this study. The transposase in the kit is a variant of the Tn5 transposase[1] and has been characterized for high throughput sequencing applications[2]. We noticed a clear preference in binding to the circularization adapter. This is highly undesirable as those fragments will not produce the information incorporated in the library. Roughly, the average length of the dsDNA circles to be prepared for sequencing is 1500 bp for targets of 3000 bp, assuming a uniform distribution. The adapter is 46 bp, and digestion inside the adapter should by chance happen in approximately 2-4% of the sequences. Instead 12% of the sequences begin with the circularization adapter, and the pattern of digestion is clearly non-stochastic (Figure S4). The reported recognition sequence for Tn5 is A-GNTYWRANC-T [1] and if we align this motif to our adapter, the best match (82%) is indeed the highest noted insertion site (position 22, Figure S4). This is contrasted to a previously noted low bias of transposition. When we compare to a human shotgun sample generally a low bias is seen, as is the case for all the raw data in this study (Figure S5). Although a general low bias is seen per position on a global scale, particular sequences can be quite strongly biased.

Supplementary tables

Table S1. Tile-seq throughput.

The theoretical output is based on a typical amount of reads obtained from one lane of Illumina HiSeq2000 sequencing, one of the 2x100 bp reads is used for classifying the construct (reading indices). The current throughput is approximated based on the mapped non-duplicated reads of the data, average read length and average amount of bases that could be generated in a typical run.

	Theoretical Throughput	Current throughput
**Reads**	150000000	255000
**read length**	100	45
**bases decoded**	15000000000	11475000
**amplicon length**	3000	3000
**amplicons at 1x**	5000000	3825
**amplicons at 30x**	166667	128
**amplicons at 100x**	50000	38

Table S2. Comparison of Tile-seq and Sanger sequencing

Cost comparison between Tile-seq and Sanger sequencing. This is a simplified example of a sequencing project containing 96, 3kb amplicons, either sequenced by Tile-seq as described in this report, or by 600 bp Sanger reads in both directions. This example is included to provide an overview of the protocol as compared to the established protocol of Sanger sequencing. Polymer consumption of Sanger sequencing is not considered in this example. If the same amplicon is prepared, 107 oligos (96 forward, 1 reverse, 2 universal and 8 adapters) is needed for Tile-seq and 8 for Sanger (each used as sequence primers). This example assumes low complexity samples (e.g. two alleles), known reference sequence within the amplicon, and unproblematic sequence (e.g. no long homopolymer stretches). If these criteria would not be fulfilled, cloning or sequence primer iterations will be required for Sanger but would not impact the Tile-seq protocol. Tile-seq would produce approximately 40x average coverage, and the Sanger protocol is used to read five 600 bp regions in both directions (2x). The manually intensive steps of Tile-seq is the first PCRs, and for Sanger sequencing setting upp the Big Dye reactions and the subsequent ethanol preparations (960 in total). The purifications of Tile-seq, in total 244, are automated and cost approximately $0.1 per reaction.

	Tile-seq				Sanger			
	Type of reaction	Number of reactions	Price per reaction	Total price	Type of reaction	Number of reactions	Price per reaction	Total price
	PCR I	96	$1.00	$96.00	PCR	96	$1.00	$96.00
	PCR II	96	$1.00	$96.00	Big Dye*	960	$15.00	$14,400.00
	USER	1	$5.00	$5.00				
A								
U	Exo III	1	$6.00	$6.00				
T	S1	1	$0.50	$0.50				
O	End Repair	8	$1.00	$8.00				
M	Methyltransferase	8	$0.40	$3.20				
A	Ligation	8	$5.00	$40.00				
T	Exonucleases	8	$2.00	$16.00				
E	EcoRI	8	$0.10	$0.80				
D								
	Circularization	1	$12.00	$12.00				
	Exonucleases	1	$2.00	$2.00				
	Nextera	1	$150.00	$150.00				
	PCR	1	$1.00	$1.00				
	Lane HiSeq2000	1	$1,250.00	$1,250.00				
	Total			$1,686.50				$14,496.00
	Coverage			40 x				2 x

Prices estimated from list prices of each respective supplier used in the manuscript unless stated below

* Price estimated from list price at Life Technologies

Table S3. Lambda Primers

ID-tag	Name	Primer
AGCTGATG	lbd_fo_1;	TGCGTCGAGAGCTCAGCCAGAGCTGATGCGACCTCGCGGGTTTTCGCT
TATCGATG	lbd_fo_2;	TGCGTCGAGAGCTCAGCCAGTATCGATGATCTCAGTGCGCTGCTGGCG
ATGCGATG	lbd_fo_3;	TGCGTCGAGAGCTCAGCCAGATGCGATGCTGCAGTCCCGGATGGACGC
ACGTCATG	lbd_fo_4;	TGCGTCGAGAGCTCAGCCAGACGTCATGGGGAGGCGCTGTGGCTGATT
TCATGTCG	lbd_fo_5;	TGCGTCGAGAGCTCAGCCAGTCATGTCGTTGCCCTGAAACTGGCGCGT
TAGCGTCG	lbd_fo_6;	TGCGTCGAGAGCTCAGCCAGTAGCGTCGATTGGCGGGGCTGTTGGTGG
TCTACTCG	lbd_fo_7;	TGCGTCGAGAGCTCAGCCAGTCTACTCGTGGTGTGGCGCAGATGCTGG
ATGACTCG	lbd_fo_8;	TGCGTCGAGAGCTCAGCCAGATGACTCGCGGTCGGGCGAGCGATGATG
ATCTATCG	lbd_fo_9;	TGCGTCGAGAGCTCAGCCAGATCTATCGTGCAGCTTCCTCGGCAACGG
ACAGATCG	lbd_fo_10;	TGCGTCGAGAGCTCAGCCAGACAGATCGCGTTTCTGCAAGCTTGGCTGTATAGT
ATACTGCG	lbd_fo_11;	TGCGTCGAGAGCTCAGCCAGATACTGCGTGACCGTAGGACTTTCCACATGCAG
TATATGCG	lbd_fo_12;	TGCGTCGAGAGCTCAGCCAGTATATGCGTCACCAACTCGTTGCCCGGT
TGCTCGCG	lbd_fo_13;	TGCGTCGAGAGCTCAGCCAGTGCTCGCGACATGCCGATTGCCAGGCTT
ATCGCGCG	lbd_fo_14;	TGCGTCGAGAGCTCAGCCAGATCGCGCGAGCGTCGACGGCTTCACGAAA
TAGTAGCG	lbd_fo_15;	TGCGTCGAGAGCTCAGCCAGTAGTAGCGTTTGGGGGCGATCGTGAGGC
AGATAGCG	lbd_fo_16;	TGCGTCGAGAGCTCAGCCAGAGATAGCGCCCGCTCTTACACATTCCAGCCC
TGTGAGCG	lbd_fo_17;	TGCGTCGAGAGCTCAGCCAGTGTGAGCGATGGCATGGTCGCTGGCTGG
TCACAGCG	lbd_fo_18;	TGCGTCGAGAGCTCAGCCAGTCACAGCGGGCTGCTTAATGGCGGTGGCT
ACTGTACG	lbd_fo_19;	TGCGTCGAGAGCTCAGCCAGACTGTACGCAACAGGCGCCGGACGCTAC
	lbd_re_1;	GTCCCCTGCGTCGCTGTGTC
	lbd_re_2;	TCAGCCTGCGAAGCAGTGGC
	lbd_re_3;	CCCTTTCAGCGGCGACGGTT
	lbd_re_4;	GGGCCAAAGCGGACACCTCC
	lbd_re_5;	ATCTGCCCGTTCGTGCCGTC
	lbd_re_6;	CTTCGCTTCGCGCGGGGTAT
	lbd_re_7;	GCTGAGGCCAATACCCGCGA
	lbd_re_8;	CCGCATCCTCAAGCGCGACA
	lbd_re_9;	TGGGTGACGATGTGATTTCGCC
	lbd_re_10;	TGAACCACAGATTCAAGTGGACGATG
	lbd_re_11;	CAAGGCGGCGTCAGCCAAGT
	lbd_re_12;	TGGTGGCGAAAGCAGAAGCA
	lbd_re_13;	GGCCGCCTGAGTGCGGTTTT
	lbd_re_14;	GGAAGCAGAACGCGCCGACT
	lbd_re_15;	TTGTCGCGCCAATCGAGCCA
	lbd_re_16;	TGCCCGTCGCTTTTGCTCCA
	lbd_re_17;	AGATGAACGTGTCCGCGCCT
	lbd_re_18;	CAGCGGTGTCTGCCAGTCGG
	lbd_re_19;	CGGATCACCGGAAAGGACCCG
	outer_U	TGCGUCGAGAGCTCAGCC*A*G

Table S4. TP53 and Canine-primers

ID-tag	Name	Primer
**Canine Mitochondria**		
ACGAGACG	mtDogVU1_fo7;	CGACGTAAAACGACGGCCAGTNNNNNNNNNNNNNNACGAGACGCCGGCCCCTTAGCCAATGCC
	mtDogV_re7;	CACACAGGAAACAGCTATGACCATGATCCCGTGGGGGTGTGGCTT
**P53**		
ATGTGTAG	p53_VU1_fo;	CGACGTAAAACGACGGCCAGTNNNNNNNNNNNNATGTGTAGCAGCCAGACTGCCTTCCG
ACTCGTAG	p53_VU2_fo;	CGACGTAAAACGACGGCCAGTNNNNNNNNNNNNACTCGTAGCAGCCAGACTGCCTTCCG
TGCAGTAG	p53_VU3_fo;	CGACGTAAAACGACGGCCAGTNNNNNNNNNNNNTGCAGTAGCAGCCAGACTGCCTTCCG
TGATCTAG	p53_VU4_fo;	CGACGTAAAACGACGGCCAGTNNNNNNNNNNNNTGATCTAGCAGCCAGACTGCCTTCCG

p53_V_re;	CACACAGGAAACAGCTATGACCATGTGCCCCAATTGCAGGTAAAAC

General Primers

Out-VU-fo	CGACGUAAAACGACGGCCA*G*T
Out-V_re	CACACAGGAAACAGCTATGACCA*T*G

*=phosphorothioate modification

Table S5. Circularization adapter design

TES-HP5-B_TPT1	GTGACTACCGAAAGGGTTTGAATTCCGACTTTTTGTCGGAATTCAAACCCTTZCGGTAGTCACT
TES-HP5-B_TPT2	GTCTGAACCGAAAGGGTTTGAATTCCGACTTTTTGTCGGAATTCAAACCCTTZCGGTTCAGACT
TES-HP5-B_TPT3	GTTGACACCGAAAGGGTTTGAATTCCGACTTTTTGTCGGAATTCAAACCCTTZCGGTGTCAACT
TES-HP5-B_TPT4	GTACTGACCGAAAGGGTTTGAATTCCGACTTTTTGTCGGAATTCAAACCCTTZCGGTCAGTACT
TES-HP5-B_TPT5	GTGCATACCGAAAGGGTTTGAATTCCGACTTTTTGTCGGAATTCAAACCCTTZCGGTATGCACT
TES-HP5-B_TPT6	GTCGTAACCGAAAGGGTTTGAATTCCGACTTTTTGTCGGAATTCAAACCCTTZCGGTTACGACT
TES-HP5-B_TPT7	GTTAGCACCGAAAGGGTTTGAATTCCGACTTTTTGTCGGAATTCAAACCCTTZCGGTGCTAACT
TES-HP5-B_TPT8	GTATCGACCGAAAGGGTTTGAATTCCGACTTTTTGTCGGAATTCAAACCCTTZCGGTCGATACT

Z=internally biotinylated T

Table S6. Nextera Amplification Primers

IL-NextAdaptor1	AATGATACGGCGACCACCGAGATCTACACGCCTCCCTCGCGCCATCAG
nxt-idx6	CAAGCAGAAGACGGCATACGAGATCG[ATTGGC]GTCTGCCTTGCCAGCCCGCTCAG

[index]=could be varied for multiplexing purposes

Table S7. TP53 Sanger sequencing primers

P53_1_fo	GGTGAGGATGGGCCTCCGGT
P53_1_re	AGGCAGGCGGATCACGAGGT
P53_2_fo	AACCCACAGCTGCACAGGGC
P53_2_re	TTAGCCAGGCATGGTGGTGC

Supplementary figure legends

Figure S1. Unidirectional exonuclease III degradation. 1% agarose gel analysis of exonuclease III degradation of differently prepared constructs. 1. Unidirectional degradation expected by uracil incorporation and subsequent USER treatment. 2. Same construct as 1 but no USER treatment (degradation in both ends expected). 3. Uracil incorporated in both ends and USER treatment (no degradation expected). 4. SacI restriction site incorporated in 1 end and SacI treatment (unidirectional degradation expected).

Figure S2. Time-point tag occurrence after mapping to the lambda amplicon references. The mapped position of each respective TP-tag detected of each amplicon is plotted using a Gaussian kernal density approximation (R). Plots indicate amplicon dependent variation but generally a distinguished order. TP8 omitted due to generally low detection levels.

Figure S3. Frequency (y-axis) of reads beginning with the adapter in the raw data, counted per starting position (x-axis) in the adapter. The plot shows a transposase

induced bias in adapter fragmentation. Dots indicate variable positions due to the TP-tag, and the last 11 bp were not included in the query due to the rise of unspecific hits.

Figure S4. Weblogo plots on the base distribution of the 12 first bases of raw data from Nextera prepared libraries. Left and middle shows a typical human shotgun sample with expected random distribution, right shows Tile-seq data. Left plot shows probability over base position, and middle and left shows bits (0.0-0.3) over base position. All plots indicate slight bias supporting the recognition sequence of Tn5.

Figure S1

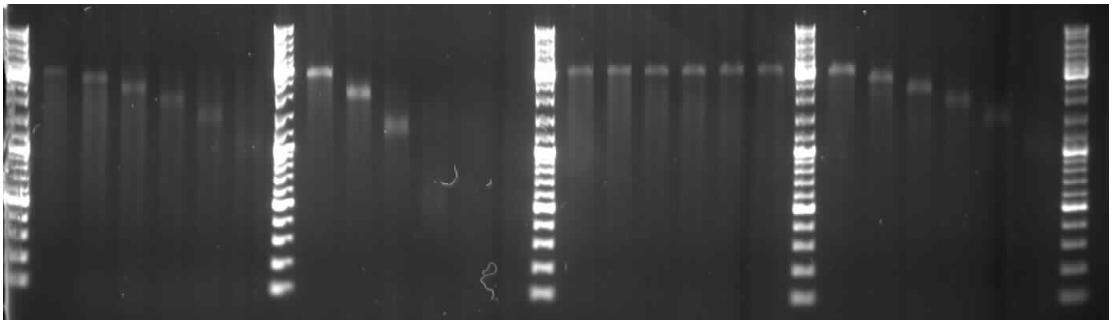

1 2 3 4

Figure S2

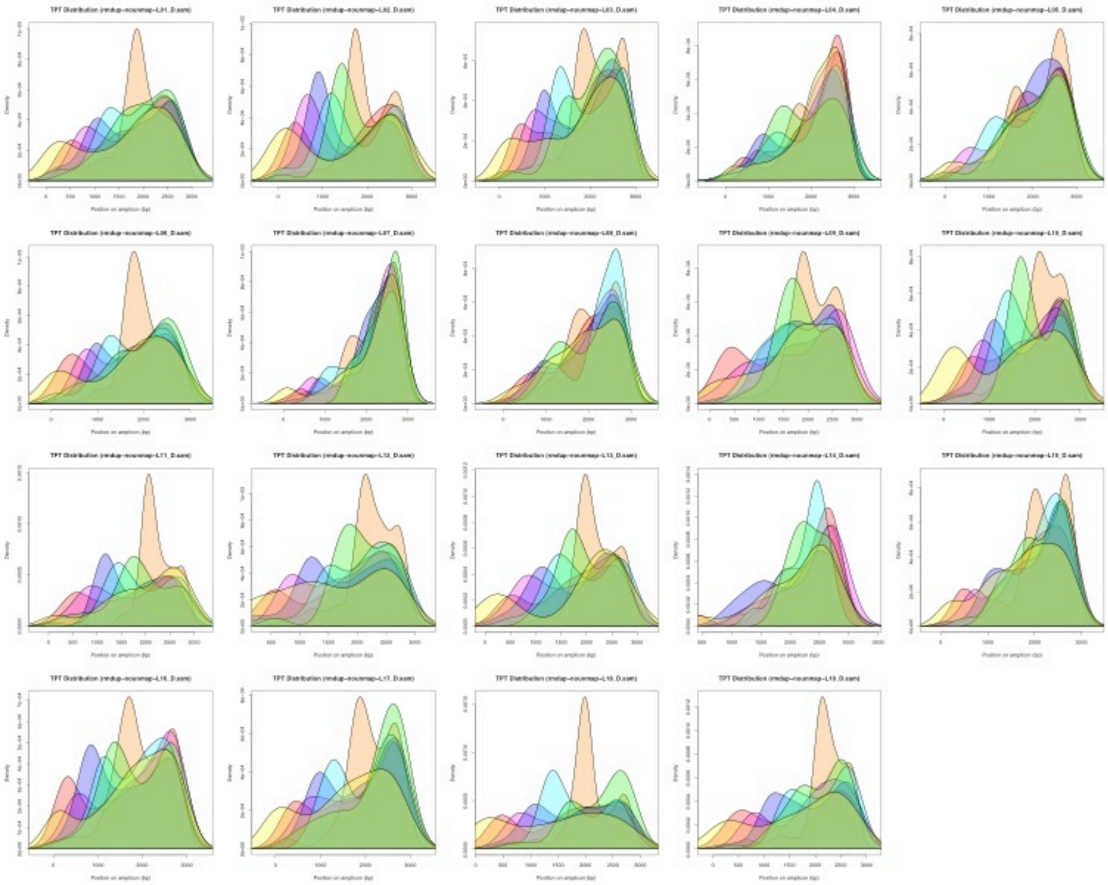

Figure S3

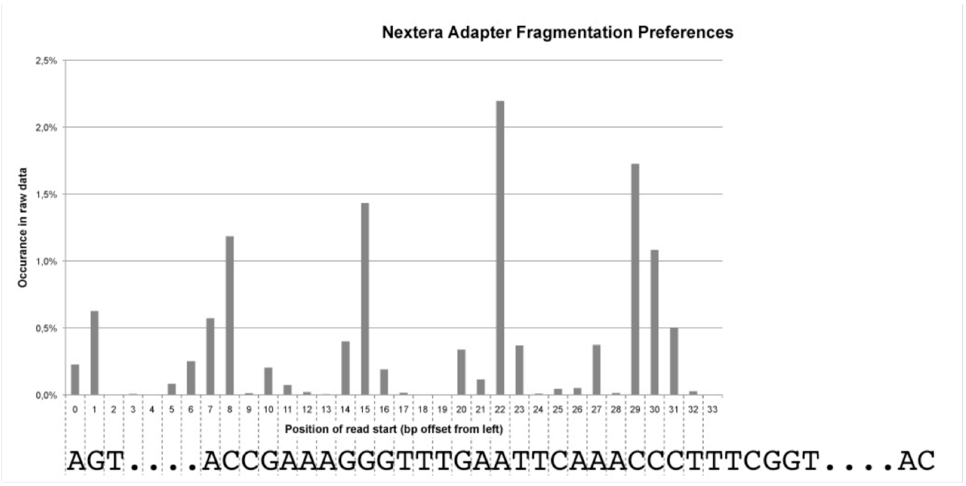
Nextera Adapter Fragmentation Preferences
Occurance in raw data
2,5%
2,0%
1,5%
1,0%
0,5%
0,0%
0 1 2 3 4 5 6 7 8 9 10 11 12 13 14 15 16 17 18 19 20 21 22 23 24 25 26 27 28 29 30 31 32 33
Position of read start (bp offset from left)
AGT....ACCGAAAGGGTTTGAATTCAAACCCTTTCGGT....AC

Figure S4

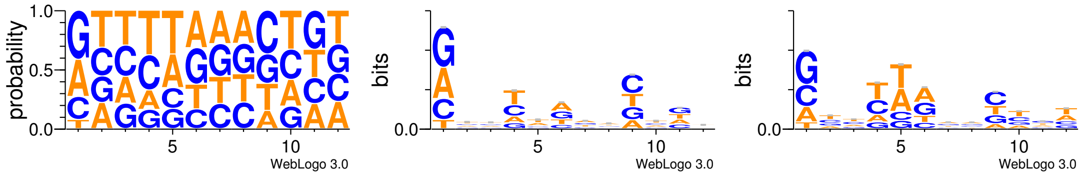

1. Goryshin, I.Y., Miller, J.A., Kil, Y.V., Lanzov, V.A. & Reznikoff, W.S. Tn5/IS50 target recognition. *Proc Natl Acad Sci U S A* **95**, 10716-21 (1998).
2. Adey, A. et al. Rapid, low-input, low-bias construction of shotgun fragment libraries by high-density in vitro transposition. *Genome Biol* **11**, R119.
